# The Holistic Review on Occurrence, Biology, Diagnosis, and Treatment of Oral Squamous Cell Carcinoma

**DOI:** 10.7759/cureus.30226

**Published:** 2022-10-12

**Authors:** Samer M Alsaeedi, Sadhna Aggarwal

**Affiliations:** 1 Molecular and Cellular Biology, Baylor College of Medicine, Houston, USA; 2 Biotechnology, All India Institute of Medical Sciences, New Delhi, IND

**Keywords:** glucose transporters, microbiome, biomarkers, human papillomavirus, oral cancer

## Abstract

A prevalent head and neck cancer type is oral squamous cell carcinoma (OSCC). It is widespread and associated with a high death rate of around 50% in some regions of the world. We discuss the likelihood of developing OSCC and the impact of age in this review. Prior to examining the vast array of diagnostic indicators, a brief explanation of the biology of the disease is addressed. Finally, the therapeutic strategies for OSCC are listed. The complete literature for this study was compiled by searching Google Scholar and PubMed using the terms "OSCC," "oral squamous cell carcinoma," "diagnosis of OSCC," "oral cancer," and "biomarkers and OSCC." The research finds that OSCC has several critical parameters with a lot of room for additional in-depth study.

## Introduction and background

**Figure 1:**
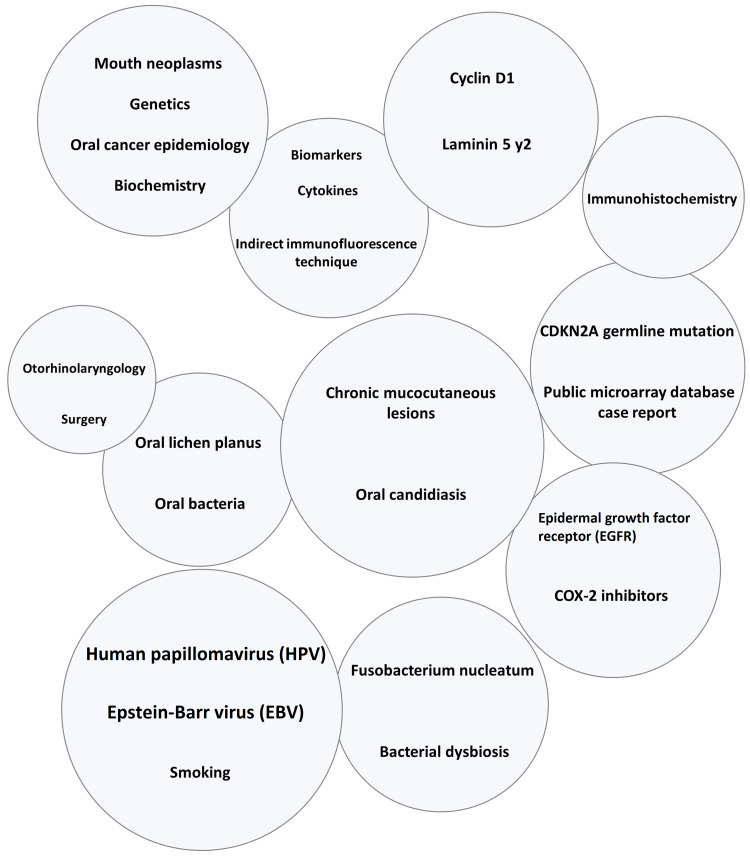
The clustering of various concepts in oral squamous cell carcinoma.

Various concepts of the most recent research in oral squamous cell carcinoma are presented in Figure 1. The Global Burden of Disease Study in the 10 most populated countries suggests trends and gender differences in the mortality rate of oral cancer [1]. The following review provides a systematic picture of the research undertaken in the fundamental concepts, which are segregated into four key sections. The first section introduces this critical issue and delves into the occurrence of OSCC and its risk influenced by age. This is followed by the biology section that gives a peek into the role of viruses and understanding the aspects of the microbiome and the bacteriome. The penultimate section deals with the current research on diagnosing OSCC via a wide range of biomarkers. The last section surmises the various treatments prescribed and the areas of interest pursued.

Occurrence and influence of age

The most common head and neck malignancy worldwide is oral cavity squamous cell carcinoma (OCSCC) [2]. It accounts for approximately 1% of cancer cases that are newly diagnosed every year in the United States [3]. On a global scale, they rank sixth among the most observed type of cancers. Roughly 90% of these cancers are histologically squamous cell carcinomas, referred to as oropharyngeal squamous cell carcinoma (OPSCC) [4,5]. To get a perspective on the enormity of this domain, after removing duplicates, 5247, 2167, and 153 articles were found across three databases, including PubMed, Scopus, and Embase.

Panda et al. used SPSS's chi-square test to compare the variations in OPSCC staging and grading between two age groups. Statistical significance was defined as a p-value of 0.05. The number or percentage of overall survival (OS), disease-free survival (DFS), recurrence, distant metastasis (DM), and second primary (SP) events in both cohorts were combined to create the odds ratio (OR), which was then used to conduct the meta-analysis. Trials were further divided into matched and mismatched studies for one or more criteria, such as age, gender, site, tumor, node, metastasis (TNM) staging, and treatments offered, in order to do subgroup analysis. The funnel plot in RevMan version 5.3 (Copenhagen, Denmark: Cochrane Collaboration) was used to evaluate publication bias. In young patients, there were 49% higher odds of recurrence in unmatched subgroup analysis and 90% higher risks of metastasis in matched subgroup analysis. Young age may be taken into account as a separate determinant of recurrence and distant metastasis (DM), according to the results, albeit additional matched studies are needed to confirm this link.

A significantly better overall survival (OS) was observed in younger patients compared to adults. The heterogeneity ranged from moderate to severe. The Surveillance, Epidemiology, and End Results (SEER) database analysis noted an increase in the average annual percentage of the incidence of oral tongue squamous cell carcinoma (OTSCC) to be more significant in men at 1.2% than in women at 0.5%, and in patients below 45 years vs. above (1.6% vs. 0.9%, respectively) from 1973 to 2010 [5]. Young patients had a non-significant tendency toward lower recurrence-free survival. Also, no appreciable difference was observed in relapse-free survival by age. According to this study, young patients with OTSCC may have a higher risk of recurrence than older patients [6]. Another important study to support this finding was the report of Garavello et al., who found the five-year DFS rates to be 34% for the young compared to 58% among the old cohorts with a p=0.003 [7].

Numerous probable causes of OTSCC's poor prognosis have been uncovered through molecular investigations. However, the study's sample size restrictions and design make it impossible to draw any definitive conclusions about age-related differences [8]. Younger patients did not have worse survival outcomes than older patients, according to a meta-analysis of nine trials (HR: 0.97; 95% confidence intervals (CI): 0.66-1.41). This study revealed that among the OCSCC patients receiving final therapy, young age is not a poor predictive survival factor due to the benefit of integrating the existing information in the systematic meta-analysis. These studies were the subject of a meta-analysis of overall survival hazard ratios, which revealed a pooled hazard ratio of 0.95. These data also imply that young individuals have comparable oncologic outcomes to older patients with a little higher age barrier. Against this backdrop, it is vital to understand the biology of the disease to probe further diagnosis and treatment.

## Review

Biology

Role of the Virus

Human papillomavirus (HPV) and OCC: Although the link between the human papillomavirus (HPV) and uterine cervix and anogenital carcinomas is well known, its role in the emergence of oral squamous cell carcinomas is still debatable. In this sense, reference lists were manually screened in the citation tracking process [9]. Studies performed on people were cohort, case-control, or cross-sectional, evaluated the HPV oncogenic activity by the E6 and E7 mRNA, contained primary oral SCC (OSCC), and/or included a biopsy to confirm the diagnosis were all considered eligible. Because none of the included research was longitudinal and none of the cross-sectional studies had a control group, it could not determine if HPV infection was related to OSCC [10].

Seventeen instances (4.4%) tested positive for HPV/mRNA. Two examples of HPV-18 and 14 cases of HPV-16 were both positive [11]. Because none of the five studies considered were longitudinal or cross-sectional and lacked a control group, it could not determine if HPV infection was related to OSCC [10]. Hence, further studies on the role of HPV infection and its relation to OSCC are paramount and could be scope for other research groups.

Role of smoking and HPV: Skoulakis et al. explored the synergistic role of smoking and the human papillomavirus (HPV) in developing cancer of the head and neck [12]. Smoking was less common in the HPV-positive group than in the HPV-negative group. So probably, there is no significant role of smoking in the pathogenesis of "head and neck squamous cell carcinoma" (HNSCC).

Maxwell et al. evaluated the role of tobacco on recurrence among HPV-positive patients who had oropharyngeal cancer (OPC) [13]. They noted a statistically significant higher risk of recurrence in current smokers than those who had never smoked. Skoulakis et al. determined that smoking is statistically more observed in HPV-negative than positive groups of HNSCC patients [12]. To fully explain the pathophysiology of HNSCC and the likely carcinogenetic pathways that are brought on by smoking and HPV, more research is, however, required.

Relationship Between Epstein-Barr Virus and OSCC

Apart from both quantitative and qualitative assessments of the Epstein-Barr virus (EBV) association with OSCC, the meta-analysis by Sivakumar et al. affirmed the association between EBV and OSCC [14]. Polymerase chain reaction, in situ hybridization, and immunohistochemistry were among the diagnostic techniques performed. Latent membrane protein (LMP)-1, EBV-determined nuclear antigen-1, and EBV-encoded small non-polyadenylated RNA-2 were among the diagnostic targets. The results of the meta-analysis revealed a connection between OSCC and EBV. However, given the several crucial limitations of the studies undertaken, there is a need for further validation of the association for any conclusive inference.

High-Throughput Nucleotide Sequencing for Bacteriome Studies

Cancer is a significant disease in modern times. Given their favorable economic and social structures and the evident aging of their populations, it is the leading cause of mortality in developed countries, particularly [15-17]. The chosen studies diverged slightly from the main goals of this review in that they used next-generation sequencing for the microbial analysis and addressed the broad topic of the connection between oral squamous cell cancer (OSCC) and microbiota.

Several articles focused primarily on comparing the oral microbiota in OSCC versus typical tissue samples [18]. Three of them had additional objectives - to make a correlation between oral cancer and certain life habits as proposed by Lee et al. [19], to analyze the genomics and metabolic pathways in microbes that are associated with OSCC [20], and to evaluate the potential growth of the bacteria's pro-inflammatory factors in their OSCC samples, chiefly by Perera et al. [20].

Most studies detected microorganisms related to inflammatory responses in the OSCC samples, like *Fusobacterium nucleatum* and *Pseudomonas aeruginosa*. While the former is linked to the OSCC of the tongue, the latter is associated with the OSCC of gingiva in addition to the tongue, for at least one study. Additionally, numerous bacteria that metabolize ethanol to create acetaldehyde, including Neisseria spp., *Rothia mucilaginosa*, and *Streptococcus mitis*, were discovered in the OSCC samples. However, the studies yielded no consensus on the hypothesis, given that often, they were found in a larger quantity within the non-tumor controls.

Association of Microbiome

The significance of microorganisms in the etiology of oral squamous cell carcinoma has garnered particular attention because periodontal disease is a microbial condition. Sami et al. offer one comprehensive review [21]. Several bacterial species have been identified in the oral squamous cell carcinoma (OSCC) samples [22]. These include relatively rare species that inhabit the oral cavity like *Bacteroides fragilis* [23], and bacteria earlier unnamed like Actinomyces and Streptococcus [24,25]. In addition, the environmental species were observed like *Dietzia psychralcaliphila* and *Gordonia sputi*. More thorough studies have been conducted on a few of these species, including *Porphyromonas gingivalis* and *Fusobacterium nucleatum*. Most haven't, nevertheless, been thought about in terms of both their singleton and polymicrobial functions in the OSCC-associated microbiome. The precise processes by which the oral microbiome may contribute to the development of OSCC are yet not fully understood [26].

Bacterial Dysbiosis - Culture-Independent Studies

According to data collected from 731 cases and 809 controls, there was no steady amelioration of any unique taxon in the oropharyngeal or oral malignancies, albeit common taxa could be distinguished between investigations. While several studies found a link between dysbiosis and oral/oropharyngeal cancer, the analytical and methodological differences made it impossible to produce a consistent summary. This emphasizes the need and scope for greater quality research with standardized methodology and reporting.

More than 30% of the non-tumor tissue included the bacteria *Granulicatella adiacens*, *Porphyromonas gingivalis*, Sphingomonas spp. PC5, and *Streptococcus mitis*/*oralis* [27]. One study reported using reagent controls to establish the lack of bacterial contamination. However, this influences the data interpretation [28]. Initial microbiome research on oral cancer has shown altered bacterial populations, including pathogens of known importance. This may indicate that bacterial genome-associated inflammatory alterations play a role in mouth cancer as a contributing factor. Identification of the specific changes in a microbe is indeed a positive step towards the development of salivary-based biomarkers of microbes in the clinical evaluation of the progress of oral cancer. 

Role of Porphyromonas Gingivalis 

OSCC is the widely observed malignant neoplasm of the oral region [29]. This study focused on the mechanisms that *P. gingivalis* plays in the development, upkeep, and/or maintenance of OSCC. In a murine model, Gallimidi et al. showed that *P. gingivalis*-infected OSCC tumors that were 4NQO-induced were noticeably more widespread and invasive, with strong expression of IL-6 [30]. The PAR4 receptor-induced over-expression of the MMP9 via kinase‐dependent signaling pathways of p38MAPK and ERK1/2. PAR2 and PAR4 were both found to be required for increasing the OSCC cell invasion potential.

*Streptococcus gordonii* and *P. gingivalis* can interact to create communities, which then colonize the tooth plaque. Because of its damaging effects on periodontal tissues, *P. gingivalis* gains from its interaction and coaggregation in the subgingival plaque [31]. The bacterium's effect in the oral epithelial cells could vary based on the phase development of OSCC, as such alterations were absent in the non-diseased gingival keratinocytes. This is further attested by the study of Liu et al. who demonstrated a novel mechanism of how *P. gingivalis *stimulates the immune evasion of OSCC via the protection of cancer from any viable macrophage attack [32]. The most recent research highlight the simplicity of managing periodontal disease (PD) as the necessary means of preventing OSCC. However, more research on human subjects is required to estimate the actual oncogenic risks from the infection of *P. gingivalis* in oral malignancy. This could also expand the scope of OSCC development, including determining the tumor's location and stage.

Biomarkers

Proteomic Markers

The neck and head squamous cell cancer (HNSCC) is a highly prevalent malignancy linked to chewing tobacco. Over the last two decades, researchers have discovered an increasing number of HNSCC patients with positive human papillomavirus (HPV) tumors that appear in younger people and those who consume less or no alcohol or tobacco. The relationship in the oropharynx is more vital than that in the oral cavity [33]. These articles were divided into subsections listed below, followed by a list of all detected protein biomarkers and a brief explanation of their significance. Clinical applications of biomarkers include detecting, diagnosing, and monitoring disease activity and evaluating therapy efficacy. Tung et al. reported the reduction of vitamin D-binding protein in OSCC plasma, suggesting differential regulation across different species [34].

Role of Glucose Transporters

The solute carriers' major facilitator superfamily has approximately 400 members, including glucose transporters (GLUTs) [35]. The distribution of glucose and other hexoses to metabolically active cells depends critically on the control of the expression of glucose transporter proteins. Two significant proteins in this class are glucose transporters 1 (GLUT-1) and glucose transporters 3 (GLUT-3) [36]. GLUT-1 expression in the Tca8113 and CAL27 cell lines was significantly higher than that in the normal oral keratinocytes (NOK) cell line, natural killer (NK). No matter if the tumor was in an early or late stage or whether it had a low or high tumor grade, GLUT-3 expression was always excessive in the deep invasive front [37]. Accordingly, there seemed no link between GLUT-3 and tumor grade [38].

GLUT-3 is the second largest researched transporter, albeit with limited research. Mixed results were found from mRNA investigations when cell lines expressed GLUT-3 [39], and frequently overexpressed in oral squamous cell carcinoma (OSCC) tumors than the adjoining healthy tissues [40,41], with occasional exceptions [42]. GLUT-1 and maybe GLUT-3 are the only two glucose transporters extensively examined in OSCC and healthy oral keratinocytes. In a different investigation by Kunkel et al., the positive cell proportion was more accurate at predicting the prognosis than the intensity of GLUT-1 staining [43]. Compared to those with cell positivity of >50%, those with cell positivity at 50% demonstrated a median survival of 138 months (p=0.0034). Clinical decision-making may benefit significantly from a greater understanding of these proteins' connections to illness development, resistance to treatment, and prognosis.

Prognostic Biomarkers

It is crucial to find accurate prognostic biomarkers for detecting oral tongue squamous cell carcinoma (OTSCC) to predict the tumor's behavior more accurately and direct the subsequent therapy decisions. There were 174 investigations carried out during the previous three decades, and 184 biomarkers were assessed for the prognostication of OTSCC. Numerous biomarkers have been proposed as helpful prognosticators for OTSCC, but the methodology and reporting quality of the original studies is generally subpar, making it impossible to draw definitive conclusions. OTSCC is increasing in incidence and has an aggressive clinical behavior with a relatively poor prognosis [44,45]. When a biomarker proved to be statistically "non-significant" in an unadjusted analysis, it was typical to reject it from an adjusted analysis using Cox regression. In contrast, biomarkers that were "significant" in an unadjusted study were frequently included in an adjusted analysis. Numerous immunohistological indicators examined in OTSCC and buccal cancer samples did not predict survival in OTSCC, although some did in buccal carcinoma [45-47].

Malondialdehyde - Oxidative Stress Marker

Squamous cell carcinoma (SCC) is an oral malignancy widely observed. The endogenous formation of malondialdehyde (MDA) during lipid peroxidation is an appropriate biomarker for endogenous DNA damage [48]. The degree of tissue damage caused by oxidative stress may be determined by estimating the lipid peroxidation by-products in the OSCC group. The research typically revealed a prominent increase of malondialdehyde in OSCC-positive cohorts than in the control healthy group. Nevertheless, to ascertain that MDA is a potential biomarker for oxidative stress and a valid prognostic marker of OSCC, this calls for a study of a grander scale with controls more evenly-balanced and equidistribution samples between the various histological grades and clinical stages of OSCC.

CircRNAs

Circular RNAs (CircRNAs), a newly discovered non-coding RNA, have been linked to carcinogenesis, metastasis, and cancer progression. They may be potential biomarkers for detecting OSCC. The post-test probability of the circRNAs was calculated using Fagan's nomogram [42]. The post-test probability increased to 47% from 20% with a positive likelihood ratio of 4 and decreased to 8% with a negative likelihood ratio of 0.33. Accordingly, it can be suggested that circRNAs are an effective and reliable diagnostic biomarker. Multiple studies have shown that dysregulated circRNAs are crucial for cancer cell proliferation, metastasis, and incidence. Compared to those who used tissue samples to diagnose OSCC patients, the use of plasma and saliva specimens demonstrated a better efficacy, with no heterogeneity.

Histopathological Features

In a crucial study, the criteria for exclusion of studies included - alternative tumors other than OSCC [49], samples that contained biopsies [50,51], immunohistochemistry-based investigations [51], histological grading systems used for analysis [52], reports of univariate survival analysis [53], studies based solely on association analysis [54], omitted the hazards for OS (HR) and/or its 95% confidence interval (CI), and reviews of associated literature [55], conference abstracts and letters [56]. During the title and abstract screening, 2490 research were included. Of these, 2074 studies were eliminated, leaving 416 studies that satisfied the requirements for full-text screening. A promising biomarker should be precise, quantifiable, relevant, accessible, and affordable. Even though this is a rapidly evolving field with standard practice for some cancers, the therapeutic approach to OSCC and its prognosis still rely on tumor, node, metastasis (TNM) clinical staging. It was useful to probe the review of the impact of histopathological traits on hematoxylin and eosin (HE)-stained slides as prognostic indicators for OSCC patients.

Perineural invasion (PNI) and disease-specific survival (DSS) were significantly correlated in a meta-analysis of 7523 individuals from 26 studies. Depth of invasion (DOI) in OSCC has only been the subject of one previous meta-analysis. Regardless of the cutoff point, this research found substantial risks of the metastasis of the lymph node during diagnosis with recurrence in tumors possessing high DOI [57].

CAIX Expression

One of the most challenging situations for the cellular and extracellular matrix to maintain homeostasis is hypoxia. Much research has investigated the prognostic value of carbonic anhydrase IX (CAIX) in varying cancer types, including OSCC [58]. The PECO framework-based investigation into the predictive significance of tumoral CAIX immunohistochemistry expression in patients with OSCC is a significant article in this field.

The analysis returned the pooled hazard ratios (HRs) doubly higher for the Asian group (HR: 1⁄4 2.01, 95% confidence intervals (CIs): 1.42-2.86) than the non-Asian group. Here, the correlation between CAIX overexpression to worsen OS and disease-free survival (DFS) in OSCC patients was confirmed, indicating a positive test implied the overall risk of mortality growing by around 50%.

S100 Proteins

Oral cancer is a significant health issue among the general public [59]. To review the literature in this domain, a detailed search was strategized for every database with free text words and the MeSH (Medical Subject Headings) combinations. The findings showed that significant increase in the levels of S100A7 in three studies [60-62].

In comparison, overexpression was reported for S100A2 [60,62], A9 [63,64], and A12 in oral squamous cell carcinoma (OSCC) patients compared to the control of healthy cohorts [65,66]. In contrast, the quantitative analysis demonstrated under expression of S100A8 [62,67], A9 [62,67], and A14 in two studies each in OSCC patients as against healthy subjects [62,66]. It is noteworthy that all studies report the overexpression of S100A7 in OSCC patients, unlike healthy individuals [61-63]. Accordingly, it is postulated that increased S100A7 protein expression is linked to the onset of oral cancer, making the protein secreted a potential OSCC biomarker.

Unfortunately, the sample size overall for the studies was small and held a significant influence on the interpretation of the findings. It is yet unclear whether certain S100 protein members' up- or down-regulation acts as a diagnostic sign in OSCC.

CYFRA 21-1 and MMP-9 as Salivary Biomarkers

Numerous techniques and tests can identify OSCC. In patients presenting with clinically obvious lesions, the diagnostic accuracy of several methods, including oral cytology, vital staining, oral spectroscopy, and light-based detection, has been assessed by a Cochrane systematic review in a dental environment [68]. When the techniques of participant recruitment are ignored, studies that compare changed expressions of a particular salivary biomarker between healthy "control" participants and "cases" with OSCC may produce false results [68].

Only six studies reported the diagnostic accuracy of detecting OSCC using salivary biomarkers cytokeratin 19 fragments (CYFRA 21-1) [69,70] and matrix metalloproteinase 9 (MMP-9) were included [71-73]. Also, one group was recruited for its study, a sample of OSCC patients and healthy or low-risk potentially malignant disorders (PMDs) cases [73].

CD68 and CD163 Tumor-Associated Macrophages

One common neoplasm in humans is squamous cell carcinoma of the head and neck (SCCHN) [74]. An important study based on the following criteria for the qualitative and quantitative analysis was conducted: (i) prospective/retrospective cohort studies that analyzed the cluster of differentiation (CD)68^+^ and/or CD163^+^ tumor-associated macrophages (TAMs) expressed in clinical dissections of SCCHN; (ii) minimum population of 20 patients in each study; (iii) semiquantitative determination using immunohistochemistry (IHC); (iv) studies that determined the TAM correlation to patients' prognosis on at least one of these parameters - disease-free survival (DFS), overall survival (OS) and progression-free survival (PFS).

While CD68^+^ TAMs were assessed in 12 studies, eight studies were analyzed for their CD163^+^ TAMs. Notably, four of these studies evaluated both TAM subpopulations [75-77]. The meta-analysis demonstrated the excess CD163^+^ TAM and negligible CD68^+^ TAM, correlating to the poor survival of HNSCC patients. In accordance with previous observations of other immunological indicators, including the programmed cell death ligand 1 (PDL1), both TAMs were more frequently expressed in females than in males [78,79]. Here, it was shown that stromal CD163^+^ TAMs are associated with a worse prognosis in SCCHN patients.

Treatment

The disease stage, location, and the patient's general health status affect how OSCC is treated. An extensive evaluation of the various treatment techniques is given by Gharat et al. [80]. Inhibitors of the epidermal growth factor receptor (EGFR) and cyclooxygenase-2 (COX-2) enzymes, photodynamic therapy, chemoprevention, nanocarrier-based drug delivery technology, polymeric nanoparticles, nanoemulsion, solid lipid nanoparticles, nanolipid carriers, carbon nanotubes, nanoliposomes, metallic theranostic nanoparticles, hydrogels, cyclodextrin based system, liquid crystals, and surface-engineered particulate system are among them.

The majority of oral squamous cell carcinoma (OSCC) cancers overexpress the epidermal growth factor receptor (EGFR/ErbB1/HER1), and links have been made between higher expression levels and an aggressive phenotype, a poor prognosis, and resistance to anticancer therapy [81]. In OSCC, prostaglandin E2 (PGE2) release is promoted by COX-2 overexpression. This stimulates the cell surface receptors (EP1, EP2, EP3, and EP4) to encourage OSCC development [82]. Accordingly, EGFR and COX-2 inhibitors have been probed as potential therapeutics.

The existing treatment modalities have brought about the main issues relating to non-specific cell death for OSCC, such as chemotherapy, radiation, invasive surgery, and photodynamic therapy. As a result, surface engineering has recently made it possible for scientists to create a variety of nanoparticles with the necessary targeting, programmed-release, and imaging properties, thus advancing the field of nano-theranostics.

Despite all these benefits, additional research is required to determine nanotechnology's practical application and efficacy for OSCC management. There aren't many studies on the direct site for treating OSCC using the nanoparticulate method via the oral cavity or the buccal mucosa. Researchers in this sector have the chance to investigate nanoparticulate systems further to enhance medicine delivery and patient quality of life.

## Conclusions

The review of the current literature on OSCC provides a clear insight into the current standing in the domain. While demonstrating the necessity for deeper exploration, our review notes the holistic aspects of this issue. The influence of age, smoking, and other viruses is detailed, while the hypotheses yet to be affirmed in the different aspects of the disease are delineated. An exhaustive account of the biomarkers used for OSCC diagnosis is discussed, outlining their comparative challenges and successes. In conclusion, this exercise strives to highlight the frontiers of the research on OSCC and the incumbent lacunae that need to be resolved.
